# High-Throughput Phenotyping Enabled Genetic Dissection of Crop Lodging in Wheat

**DOI:** 10.3389/fpls.2019.00394

**Published:** 2019-04-03

**Authors:** Daljit Singh, Xu Wang, Uttam Kumar, Liangliang Gao, Muhammad Noor, Muhammad Imtiaz, Ravi P. Singh, Jesse Poland

**Affiliations:** ^1^Interdepartmental Genetics, Kansas State University, Manhattan, KS, United States; ^2^Department of Plant Pathology, Kansas State University, Manhattan, KS, United States; ^3^Borlaug Institute for South Asia, Ludhiana, India; ^4^Department of Agriculture, Hazara University, Mansehra, Pakistan; ^5^International Maize and Wheat Improvement Center, Islamabad, Pakistan; ^6^Global Wheat Program, International Maize and Wheat Improvement Center, Texcoco, Mexico

**Keywords:** *Triticum aestivum*, GWAS, genomic selection, high-throughput phenotyping, lodging, UAV/UAS, unmanned aerial systems, wheat breeding

## Abstract

Novel high-throughput phenotyping (HTP) approaches are needed to advance the understanding of genotype-to-phenotype and accelerate plant breeding. The first generation of HTP has examined simple spectral reflectance traits from images and sensors but is limited in advancing our understanding of crop development and architecture. Lodging is a complex trait that significantly impacts yield and quality in many crops including wheat. Conventional visual assessment methods for lodging are time-consuming, relatively low-throughput, and subjective, limiting phenotyping accuracy and population sizes in breeding and genetics studies. Here, we demonstrate the considerable power of unmanned aerial systems (UAS) or drone-based phenotyping as a high-throughput alternative to visual assessments for the complex phenological trait of lodging, which significantly impacts yield and quality in many crops including wheat. We tested and validated quantitative assessment of lodging on 2,640 wheat breeding plots over the course of 2 years using differential digital elevation models from UAS. High correlations of digital measures of lodging to visual estimates and equivalent broad-sense heritability demonstrate this approach is amenable for reproducible assessment of lodging in large breeding nurseries. Using these high-throughput measures to assess the underlying genetic architecture of lodging in wheat, we applied genome-wide association analysis and identified a key genomic region on chromosome 2A, consistent across digital and visual scores of lodging. However, these associations accounted for a very minor portion of the total phenotypic variance. We therefore investigated whole genome prediction models and found high prediction accuracies across populations and environments. This adequately accounted for the highly polygenic genetic architecture of numerous small effect loci, consistent with the previously described complex genetic architecture of lodging in wheat. Our study provides a proof-of-concept application of UAS-based phenomics that is scalable to tens-of-thousands of plots in breeding and genetic studies as will be needed to uncover the genetic factors and increase the rate of gain for complex traits in crop breeding.

## Introduction

A deeper understanding of the biological processes mediated by plant genomes is needed to develop crops with improved stress resilience and yield potential. Connecting genotype to phenotype for quantitative plant traits on a genome level necessitates high-density genetic markers and large population sizes to gain sufficient power and resolution. While the recent advancements in sequencing technologies have provided almost unlimited access to high-density genetic markers, large-scale rapid and accurate phenotyping of complex traits remains a major constraint. High-throughput phenotyping (HTP) tools with improved spatial and temporal resolution can help address this phenotyping bottleneck (Furbank and Tester, 2011; White et al., 2012).

Several HTP platforms including greenhouse, ground-based, and aerial systems have been demonstrated for crops (Andrade-Sanchez et al., 2014; Honsdorf et al., 2014; Crain et al., 2016), such as enabling the dissection of stress and growth traits in controlled conditions (Chen et al., 2014; Campbell et al., 2015; McCormick et al., 2016). For targeting the scope of field experiments, unmanned aerial systems (UAS) offer a flexible alternative to ground-based phenotyping platforms, particularly for large-breeding nurseries and genetic studies with thousands or tens-of-thousands of plots (Poland, 2015). Recently, UAS have been deployed in HTP of wheat breeding nurseries (Sankaran et al., 2015; Haghighattalab et al., 2016), expanding previous work using multi-rotor UAS of varying sizes and payload capacity to phenotype small-sized test plots (Bendig et al., 2014; Chapman et al., 2014; Shi et al., 2016). With rapid development of low-cost consumer-grade sensors and platforms, UAS phenotyping holds great potential to be an integral part of plant genomics and breeding for precise, quantitative assessment of otherwise low-throughput and complex traits on large populations. However, significant developments in processing, methodology and analysis of UAS-derived data are needed to realize its full potential.

Lodging, the permanent displacement of the plant stem from vertical position, is an example of a complex trait that is difficult to quantify in the field (Pinthus, 1974). Conventional phenotyping methods for lodging are based on visual ratings of incidence and severity scores, and can be associated with stem or root lodging (Berry et al., 2003; Piñera-Chavez et al., 2016). A lodging-resistant ideotype target for wheat has been described as a strong root system, wider root plates, larger stem diameter, and moderate to short height (Berry et al., 2007). The physiology of lodging in wheat is associated with a complex genetic architecture (Verma et al., 2005; Liu et al., 2015; Miller et al., 2016). Only a few small to moderate effect quantitative trait loci (QTL) explaining 2–27% variation for lodging and stem strength have been identified (Keller et al., 1999; Hai et al., 2005; Berry and Berry, 2015). As such, the complex genetic architecture of lodging is a good target for field-based HTP to enable precise measurement of very large populations needed for genomics studies and breeding progress.

Image-derived lodging assessments have been proposed in wheat, maize, and rice (Chapman et al., 2014; Chu et al., 2017; Yang et al., 2017). Albeit on a limited number of plots and without quantitative assessment or ground-truth validations, a proof-of-concept study by Chapman et al. (2014) demonstrated the possibility to assess the presence of lodging with UAS. An image-based lodging assessment was validated relative to visual scores of lodging on 288 maize plots (Chu et al., 2017). Thus, with strong proof of concept and scalable potential, UAS assessment of lodging phenotypes in large wheat breeding nurseries has potential to transform throughput, and hence the power, for genetic studies and breeding programs.

Here, we demonstrate novel field-based lodging assessment approaches using a commercially available light-weight UAS. By developing multiple time-points of three-dimensional digital elevation models (DEM) from UAS-acquired stereo imaging, we quantified lodging in 2640 wheat breeding plots with high correlation to visual scores and comparable repeatability. Using these precise phenotypic measurements, we identified genomic regions associated with lodging in wheat from a genome-wide association analysis. The limited genetic variation explained by the genome-wide associations led us to test whole-genome prediction models which accounted for a much larger portion of the heritable variation and supported the need for large, precisely measured populations to understand the functional genomics of lodging. Here, we report an original application of UAS for large-scale, high-throughput assessment of complex plant architecture and physiology in breeding and genomic studies with evaluation of lodging in wheat. This highly reproducible approach is scalable to tens-of-thousands of plots or even individual plants of different crops to rapidly quantify plant height, lodging, and could potentially be extended to traits like growth rate.

## Materials and Methods

### Plant Material and Field Layout

Advanced spring wheat (*Triticum aestivum* L.) breeding lines from CIMMYT’s South Asia Bread Wheat Genomic Prediction Yield Trials were sown in the first week of November (Nov 4, 2015 and Nov 7, 2016) at the Borlaug Institute for South Asia’s Ludhiana (LDH), Punjab, India (30°59′ N and 75°44′ E) location during seasons 2015–2016 and 2016–2017. A total of 590 and 595 unique wheat entries along with the check varieties were planted in alpha-lattice field design during seasons 2016 and 2017, respectively. Entries in each year were divided into 11 trials with each trial containing 53 closely related entries and 7 checks laid out in two complete replicate blocks of 120 plots per trial. Each replicate block was divided into six subblocks of 10 plots each. The experimental unit was an individual six-row plot with dimensions 1.3 m × 3.8 m. Plot-to-plot spacing was 80 cm and 52 cm between ranges and columns, respectively. Sixty best entries from the 2015–2016 season were repeated in the 2016–2017 season as an additional trial. The same experiment was replicated in Faisalabad (FAS) in Pakistan (31°24′ N and 73°02′ E), without the 11th trial. The experimental location in LDH is situated in the north-western wheat growing belt of India. LDH and FAS environments are classified as irrigated mega-environments (ME1) according to CIMMYT’s wheat breeding mega-environment classification system (Rajaram et al., 1995). Field trials were managed following the established standard agronomic practices at each location.

### UAS and Sensor Specifications

Two different UAS quadcopters were deployed for data acquisition during two seasons at LDH. In 2016 season, an IRIS+ quadcopter (3DR Robotics Inc., Berkeley, CA, United States) equipped with a 3-channel Canon S100 digital camera (Canon, United States) was used to collect data over the wheat plots. In 2017, the UAS platform was upgraded to a high payload carrying capacity quadcopter DJI Matrice100 (DJI, United States) carrying a 5-channel multispectral RedEdge camera (MicaSense Inc., United States). A detailed list of UAS and sensor specifications is provided (Table 1).

**Table 1:** Experimental details of the study.

	Season 2016	Season 2017
No. unique entries	590	595
No. of plots	1,320	1,320
Plot size	1.3 × 3.8 m^2^	1.3 × 3.8 m^2^
Field design	α-Lattice	α-Lattice
Pre-lodging flight	March 01	March 02
Post-lodging flight	March 16	March 15
Ground-truth date	March 18	March 15
UAS platform	3DR IRIS+	DJI M100
In-air flight duration	25–30 min	20–25 min
Flight speed	2 m/s	2 m/s
Flight altitude	25 m	25 m
DEM resolution	1.5 cm/pixel	3.5 cm/pixel
Camera sensor	Modified Canon S100	MicaSense RedEdge
Camera bands (nm)	Blue (460), green (525), near infrared (710)	Blue (475), green (560), red (668), RedEdge (717), near infrared (840)

*UAS, unmanned aerial system; DEM, digital elevation model.*

### UAS-Based Image Acquisition

Each year the semi-autonomous UAS flights were conducted between 11AM and 2PM. Data acquisition followed the standard operating procedures developed within the Poland Lab at Kansas State University (Wang et al., 2018). In each of the years, the field trials experienced natural lodging from the combination of heavy rain and wind during the grain-filling stage. A total of four UAS flights were made on days March 01, 2016, March 16, 2016, March 02, 2017, and March 15, 2017. The 2016 and 2017 flight dates correspond to pre- and post-lodging events, respectively. The flight plans were created using Mission Planner desktop application for Windows^1^ for IRIS+ UAS, and Litchi Android App (VC Technology Ltd.) and CSIRO mission planner application^2^ (accessed October 2, 2018) for DJI Matrice100. All flights were made at a ground altitude of 25 m in 2016 and 2017. In both years, the image overlap rate between two geospatially adjacent images was set to 80% sequentially and 78% laterally to ensure optimal orthomosaic photo stitching quality. Accordingly, the flight speed, the flight elevation above the ground, and the width between two parallel flight paths were adjusted based on the overlap rate and the camera field of view. Both cameras were automatically triggered with the onboard GPS following a constant interval of distance traveled.

To ensure highly accurate digital elevation maps, the UAS images were geo-referenced and geo-rectified using 12 white colored ground control points (GCPs) that were uniformly distributed over the entire 1.5 ha field area. These GCPs were surveyed using a SXBlue III-L differential Global Navigation Satellite System (GNSS) unit (Geneq Inc., Montreal, QC, Canada) and Precis BX305 Real Time Kinematics GNSS unit (Tersus GNSS Inc., Shanghai, China) in 2016 and 2017, respectively. To preserve the image pixel intensity, the Canon S100 camera was set to capture raw images, while the MicaSense RedEdge camera was set to capture uncompressed TIFF images.

### Digital Elevation Model Generation

Raw images captured by Canon S100 camera were imported to Canon Digital Photo Professional Software (Canon, United States) for lens distortion correction and converted to 16-bit TIFF images. Lens distortion corrections were not required for images captured by MicaSense RedEdge camera^3^. After preprocessing, images of both cameras were processed in Agisoft PhotoScan Pro (Version 1.3.1, Agisoft LLC, Russia) following the internally established protocols. In the first step of image alignment, the settings were: key-point limit 15000 (MicaSense) and 80000 (Canon) points, reference pre-selection, accuracy high, tie-point limit 0, and adaptive camera model. A sparse point cloud of the entire field area was stitched through the process of image alignment. In the subsequent step, the GCP coordinates were assigned to the individual images where white-colored, square-shaped GCPs were visible on the ground. Typically, at least three images per GCP are required to accurately geo-rectify and geo-reference the orthomosaics. The GCP assignment step was followed by the camera optimization step that adjusted the estimated point coordinates and camera parameters in the model. Based on the optimized camera positions, a dense point cloud model of the entire field was generated by setting the parameters to high quality and moderate depth. Finally, the DEM was built from the dense point cloud model. The detailed processing reports are available on the project data repository (Singh et al., 2018). Each pixel in this DEM had three attributes namely latitude, longitude and height. These three attributes corresponded to geo-position and height of DEM points, and were used to calculate plot-level height/lodging information. A total of four DEM with two DEM each season corresponding to pre- and post-lodging, respectively, were generated.

### Lodging Assessment

**FIGURE 1:**
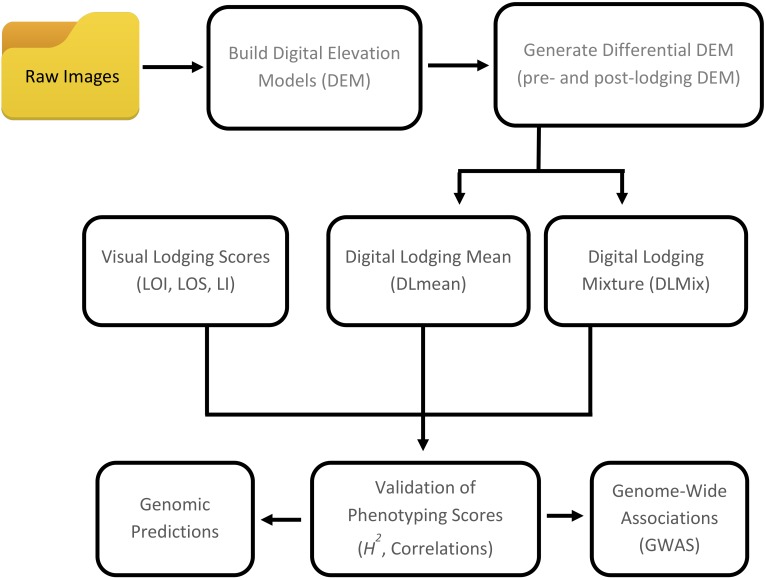
Workflow of digital and visual phenotypic analysis approaches used to assess crop lodging in wheat.

**FIGURE 2:**
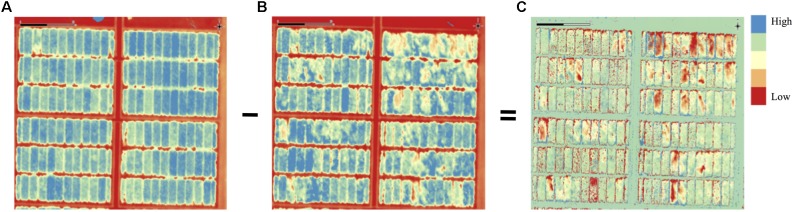
Processing of pre- and post-lodging digital elevation models (DEM) to obtain differential DEM of lodging. Post-lodging DEM is subtracted from pre-lodging DEM to generate a differential DEM of lodging. Panels are **(A)** pre-lodging, **(B)** post-lodging, and **(C)** differential DEM. Elevation differences are color coded with red corresponding to low elevation in **(A,B)** or high differences in **(C)**, blue is areas of high elevation **(A,B)** or low differences **(C)**.

In the present study, we describe two crop lodging assessment algorithms based on image-derived DEM. Both approaches used a differential DEM model that was generated by subtracting the post-lodging DEM from pre-lodging DEM each year (Figure 1, 2). In the first method, a simple arithmetic mean (DLmean; Digital Lodging mean) of the differential digital elevation pixels for each plot was calculated. In the second method, a two-component normal mixture distribution with parameters μ_2_ and λ_2_ was estimated through an iterative Expectation Maximization algorithm in *R* package *mixtools* (Benaglia et al., 2009) by constraining the mean parameter μ_1_ to zero. The parameters μ_2_ and λ_2_ correspond to the mean and proportion of the lodged DEM pixels, respectively, and were combined to create a mixture lodging index of the digital lodging (henceforth, Digital Lodging Mixture, DLmix; μ_2_ × λ_2_). Additionally, visual assessment of lodging was carried out post-lodging events in both seasons. Visual scores included the lodging intensity (LOI; percent plot area lodged; 0–100%), severity (LOS; angle of plant lodging; 0–10), and a combined lodging index (LI; LOI × LOS). Additional agronomic and phenological traits were collected at different growth stages during the season. The final grain yield was measured on per plot basis. A detailed trait description as well as the ontology information is provided (Supplementary Table S1).

### Statistical Data Analysis

The phenotypic data on lodging included: (i) three visual scores of lodging namely intensity (LOI), severity (LOS) and lodging index (LI) per plot and additional supporting agronomic measurements per plot; (ii) two digital lodging scores obtained by taking overall summary mean per plot (DLmean) or combined lodging index of normal mixture parameters (DLmix). The variance components for broad-sense line mean heritability or repeatability for each trait and trial were calculated using *lme4* package (Bates et al., 2014) in R with the following model:

(1) $$y_{ikl}=\mu +G_{i}+M_{k(l)}+e_{ikl}$$

where y_ikl_ is the phenotypic response variable, μ is the fixed overall mean, G_i_ is the random genotype effect, M_k(l)_ is the random effect of sub-blocks nested within a replicate, and e_ikl_ is the residual effect. The variance components derived from the model were used to calculate broad-sense heritability on entry-mean basis for each trait:

(2) $$H^{2}=\frac{\sigma_{G}^{2}}{\sigma_{G}^{2}+\sigma_{e}^{2}/r}$$

where *σ^2^_G_* is the genotypic variance, *σ^2^_e_* is the residual variance, and *r* is the number of replicates. Genotypic best linear unbiased estimates (BLUEs) were calculated as follows:

(3) $$y_{ijkl}=\mu +G_{i}+Z_{j}+R_{k(j)}+M_{kl(j)}+e_{ijkl}$$

where *y_ijkl_* is the phenotypic response variable, μ is the fixed overall mean, G_i_ is the random genotype effect, *Z_j_* is the random trial effect, *R_k(j)_* is the random effect of replicate nested within a trial, *M_kl(j)_* is the random effect of sub-blocks within replications nested within a trial, and *e_ijkl_* is the residual effect. The marker-based genetic correlations between each pair of traits were calculated with *sommer* (Covarrubias-Pazaran, 2016) package in R as follows:

(4) $$r_{g}(x,y)=\frac{{\mathrm{cov}}_{g}(x,y)}{\sqrt{{\mathrm{var}}_{g}(x)*{\mathrm{var}}_{g}(y)}}$$

where *cov_g_(x,y)* is the covariance of the trait pairs *x* and *y*; *var_g_(x)* and *var_g_(y)* is the variance of traits. The variance and covariance parameters of a pair of traits were estimated by fitting a multivariate model on two traits at a time.

### Genotyping

All 1,185 lines from both seasons were profiled using the genotyping-by-sequencing protocol of Poland et al. (2012) and sequenced on an Illumina Hi Seq2000 or HISeq2500. Single nucleotide polymorphism (SNP) markers were called with TASSEL *v*5 pipeline (Glaubitz et al., 2014) and aligned to the reference Chinese Spring Wheat Assembly v1.0 (International Wheat Genome Sequencing Consortium [IWGSC], 2014). Genotyping calls were extracted and filtered so that the percent missing data per marker was less than 40% and percent heterozygosity was less than 10%. Lines with more than 50% missing data were removed. After filtering, a total of 10,878 SNP markers were retained and missing data were imputed with Beagle *v*4.1 (Browning and Browning, 2016). Another filtration step was applied on imputed SNPs to remove heterozygous calls. In addition, we built a bioinformatics pipeline to predict the presence or absence of the 2NS segment based on genotyping-by-sequencing. Briefly, wheat and alien specific tags were identified using a training set of cultivars or lines that are known to be 2NS positive and negative. The presence or absence of the 2NS segment was predicted based on relative counts of wheat or alien specific tags. A custom R function that takes input of alien or wheat specific tags and tags by taxa file through TASSEL pipeline was used to predict the presence or absence of the 2NS segments. The method was validated using a wet lab method (Ventriup-LN2) and proved to be highly accurate (>99%) (Liangliang Gao, unpublished). The method predicted 1,010 from 1,185 lines that were either positive or negative for alien 2NS segments.

### Genome-Wide Association Study (GWAS)

A GWAS and genomic prediction analyses were performed on 10,166 SNPs scored on 590 and 595 lines from cropping seasons 2016 and 2017, respectively. A combined GWAS analysis on 1,035 genotypes from both years was also performed with year as a fixed effect. A two-step adjusted means model with genotypes and year as fixed terms was used to generate BLUEs for the 1,035 lines for all lodging measurements as following:

(5) $$y_{ij}=\mu +G_{i}+E_{j}+e_{ij}$$

where *y_ij_* is the phenotypic value, μ is the fixed effect for overall mean, G_i_ is the fixed genotype effect, *E_j_* is the environment fixed effect, and *e_ij_* is the residual error. The resulting adjusted means were used as response variable in the combined association analysis. A linear mixed model of GWAS was implemented (Yu et al., 2006; Kang et al., 2008):

(6) y=Xβ+Zu+Sτ+e

where *y* is a *n* × 1 vector of adjusted means (BLUEs) of phenotypes, β is a *f* × 1 vector of fixed effect terms (intercept and principal component-based population structure covariates), *u* is a *n* × 1 vector of polygenic background effects, and τ is the additive marker allele effect. The respective design matrices: *X* is a *n* ×*f* matrix where *f* is the number of fixed covariates and *n* is the number of individuals; Z is a *n* ×*n* matrix relating *y* to *u*; *S* is a *n* ×*1* vector of marker scores. The equation 6 was implemented in *rrBLUP* package in *R* (Endelman, 2011), where each marker is independently tested to estimate the effect τ (a scalar), by treating the term *S* as a column vector of marker score covariates which can take values of -1, 0, or 1. For each trait, a genome-wide false discovery rate threshold was calculated based on the QVALUE function in *R* (Storey and Tibshirani, 2003).

### Genomic Prediction and Cross-Validation

To test for a highly polygenic genetic architecture of lodging in wheat, we generated *k*-fold based genomic predictions. To reduce the prediction bias resulting from training and testing sets similarities, 11-fold training-testing composition was chosen based on total number of trials in the experiment. Each fold left a single trial out of the training set for testing the prediction. Breeding lines were grouped into trials by pedigree, therefore, this approach for cross-validation by trial prevents any full-sib progeny from being in the training and prediction set. Two linear parametric methods of genomic predictions, ridge regression BLUP [RR-BLUP (Endelman, 2011)] and Bayes Cπ (Perez and de los Campos, 2014), and a non-linear method, Reproducing Kernel Hilbert Space [RKHS (Gianola et al., 2006)], were used to calculate genomic estimated breeding values for each trait. While RR-BLUP assumes an infinite number of loci with infinitesimally small effects, the Bayes Cπ is a variable selection method that allows for a proportion of marker effects to be set to zero, assuming a common non-zero variance for rest of the marker effects (Legarra et al., 2008; Habier et al., 2013). RKHS is a kernel-based regression method. The default hyper-parameter values as described in detail in Perez and de los Campos (2014) and http://genomics.cimmyt.org/BGLR-extdoc.pdf (see Table 1 for default prior densities implemented) were used for Bayes Cπ and RKHS methods.

Two different empirical cross-validation schemes were tested for prediction models: (1) predictions across environments on same genetic material (16LDH-16FAS, 17LDH-17FAS); (2) predictions across years on independent set of lines (16LDH-17LDH, 17LDH-16LDH, 16LDH-17FAS, 17LDH-16FAS). In the case of LDH-FAS training-testing combinations, each of the five lodging measures (LOI, LOS, LI, DLmean, and DLmix) was used in the training set at LDH to predict the LOI at FAS. The genomic prediction models were implemented in *R* packages *BGLR* and *rrBLUP* (Endelman, 2011; Perez and de los Campos, 2014). We used 10,000 iterations, 3,000 burn-ins and thinning parameter of 3 for Bayes Cπ and RKHS models in *BGLR*.

### Data Availability

All data associated with the experiments including raw images, orthomosaics, polygons, etc. can be accessed at the public repository^4^. Analysis scripts are available at github.com/singhdj2/digital-lodging.

## Results and Discussion

### High Throughput Phenotyping of Wheat Breeding Trials

**FIGURE 3:**
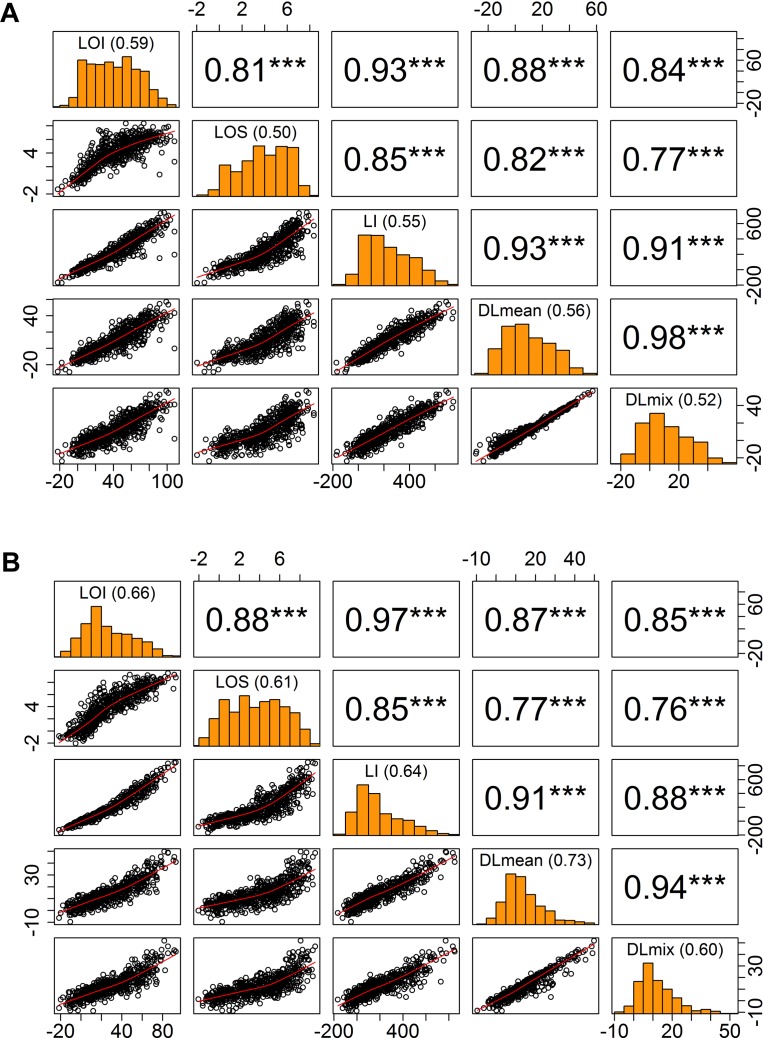
Relationship of visual and digital lodging scores. Pairwise correlation matrix of visual and digital measures of lodging in **(A)** year 2016, **(B)** year 2017. Diagonal panels show the trait distributions and broad-sense entry mean heritability; upper triangle is the Pearson’s correlation coefficient values with significance levels as superscript (^∗∗∗^*P* < 0.001); lower triangle is the scatter plot. LOI, lodging incidence; LOS, lodging severity; LI, lodging index; DLmean, digital lodging mean; DLmix, digital lodging mixture.

To assess yield potential and agronomic performance of elite breeding lines as part of the International Wheat and Maize Research Center’s (CIMMYT) breeding efforts, wheat trials were established at Ludhiana (LDH) in NW India and Faisalabad (FAS) in central Pakistan in 2016 and 2017 (Table 1). Throughout the growing season, autonomous phenotyping operations were conducted at LDH with a GPS-guided UAS equipped with modified-RGB and multi-spectral digital cameras. Mid-day flight missions covered an area of 1.5 ha containing the entire trial of over 1,320 plots in ∼25 min and obtained a ground spatial resolution of 1.5–3.5 cm per pixel. Following the natural lodging events during grain filling stage in both years, lodging incidence and severity was visually scored as a ‘ground-truth’ for subsequent validation of the image-derived lodging values (Figure 1 and Table 2). The breeding lines in the trials showed considerable phenotypic variation with 0 to 100% lodging severity and incidence, and moderate broad-sense heritability (*H^2^*) of 0.50 to 0.66 for the lodging incidence (Figure 3).

**Table 2:** Approaches to assess lodging using digital images derived from UAS and ground-based assessment.

Data	Trait	Description
Ground-truth	Lodging incidence (LOI; 0–100%)	Visual scores of lodging
	Lodging severity (LOS; 0–10)	
	Lodging index (LI; LOI × LOS)	
Image-based digital lodging	Differential mean (DLmean)	Plot summary mean
	Digital lodging mixture (DLmix)	Normal mixture-based lodging index

### Extraction of Image-Derived Digital Lodging

To quantitatively assess the amount of lodging from UAS collected images across 1,320 field plots in each of the 2 years, we built digital elevation models (DEM) for the crop before and after the lodging events. A differential DEM was generated by subtracting the post- from pre-lodging DEM giving the overall elevation change between the two time-points (Figure 2). We observed large elevation changes that were commensurate with severely lodged plots. To derive a quantitative measurement of lodging, we first calculated a simple arithmetic mean (henceforth, Digital Lodging mean: DLmean) of all differential DEM height points falling under the area of each plot polygon. This measure of lodging was phenotypically and genetically well-correlated to the visual scores of incidence, severity, and lodging index (*r_pheno_* = 0.77–0.93; *r_geno_* = 0.93–0.96; *P* < 0.001; Figure 3 and Supplementary Table S2).

Following on the simple mean difference we applied a more informed normal mixture model of the differential DEM pixel distributions. A combined mixture lodging index (DLmix) of digital lodging was derived from the mixture model parameters and compared with the visual scores. The ground-truth validation of the mixture model again showed high phenotypic and genetic correlations to the visual scores (*r_pheno_* = 0.76–0.91; *r_geno_* = 0.93–0.97; *P* < 0.001; Figure 3 and Supplementary Table S2).

As an additional measure of accuracy of the visual scores and digital image-based estimations of lodging, we calculated the broad-sense heritability (*H^2^*), or repeatability, on an entry-mean basis for each of eleven trials in both years (Figure 3). The repeatability of visual scores, DLmean, and DLmix was in the range of 0.5–0.7, consistent with previous studies on wheat and sorghum that reported similar heritability for lodging (Liu et al., 2015; Piñera-Chavez et al., 2016; Yu et al., 2016). Digital lodging outperformed visual scores in terms of heritability for 8 out of 11 trials in year 2017 (data not shown). Digital and visual measures of lodging were genetically highly correlated in both years (*r_geno_* > 0.93; Supplementary Table S2), suggesting they are capturing the same variance and supporting the effectiveness of image-based lodging assessment for HTP within large wheat breeding and genetic studies.

### Genome-Wide Association Analysis of Lodging

**FIGURE 4:**
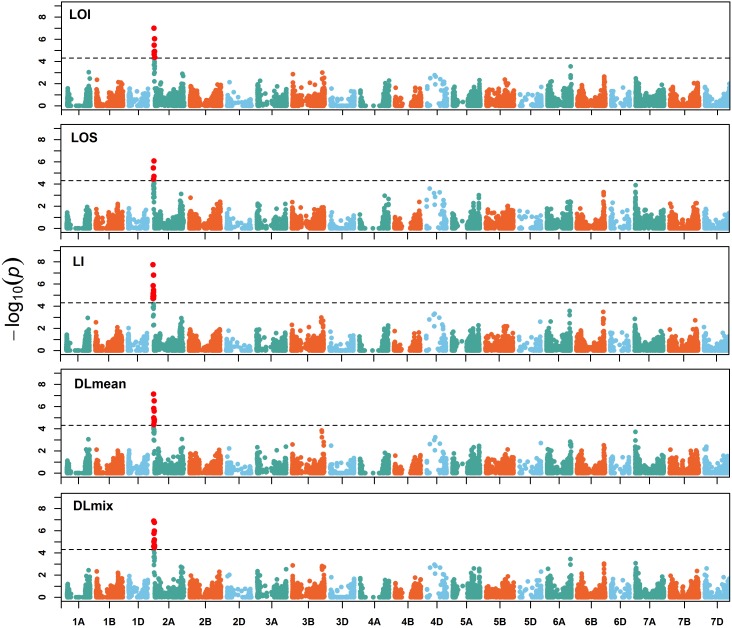
Manhattan plot of genome-wide associations. Manhattan plots of visual and digital lodging scores from combined analysis of genotypes from 2016 and 2017 (no. of genotypes = 1,035). The dashed lines on *y*-axis correspond to the genome-wide false discovery rate (FDR = 0.05) threshold. LOI, lodging incidence; LOS, lodging severity; LI, lodging index; DLmean, digital lodging mean; DLmix, digital lodging mixture.

**FIGURE 5:**
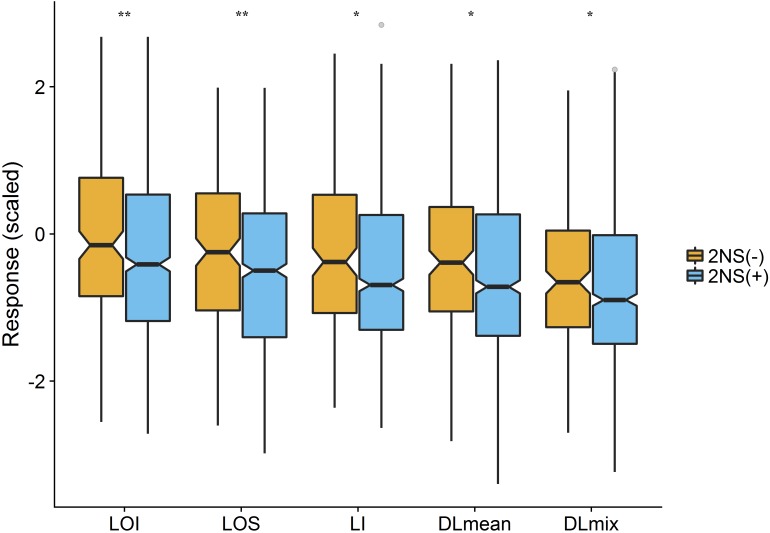
Effect of 2NS translocation on lodging. The notched boxplot of phenotypic values of lodging measures for 2NS positive (2NS+) and negative (2NS–) genotypes. The asterisks show the significant *p*-value for each trait (*t*-test; *n* = 1010*; ^∗^P* < 0.05*, ^∗∗^P* < 0.01). LOI, lodging incidence; LOS, lodging severity; LI, lodging index; DLmean, digital lodging mean; DLmix, digital lodging mixture.

To assess the genetic architecture of lodging using the validated digital image-based estimations, we conducted a genome-wide association analysis on 1,185 (590 in 2016 and 595 in 2017) elite wheat breeding lines. Genome profiling was performed with genotyping-by-sequencing and markers were fitted in a linear mixed model with terms to account for population structure and cryptic relationships (Equation 4). Despite having a relatively large population size (*n* = 590), and moderate to high heritability, no significant SNPs were identified for any lodging measure in 2016 (Supplementary Figure S1). For 2017 field trial, an association peak on chromosome 2A was observed for visual and digital scores of lodging (Supplementary Figure S2). To leverage the power of large population size, association analysis on combined data from 2 years was performed. The association test showed a highly significant and consistent peak at chromosome 2A (Figure 4 and Supplementary Figure S3). The markers on 2A coincided with a region corresponding to the 2NS *Aegilops ventricosa–Triticum aestivum* translocation (Doussinault et al., 1983; Gao et al., 2018). We investigated lines positive for 2NS translocation, which showed reduction in lodging incidence for both visual and digital lodging measures (Figure 5; *P* = 0.049–0.002, *n* = 1010*; t*-test). A survey for the 2NS fragment in our material suggested that more than 75% lines carry this translocation fragment, which is known to harbor multiple disease resistance genes in wheat (Jahier et al., 2001; Helguera et al., 2003; Williamson et al., 2013; Cruz et al., 2016). However, reports on its impact on lodging are lacking. Interestingly, the significant markers from association analysis only explained up to 2% of genetic variation for lodging and the majority of the markers were below the significance threshold. Overall, these results point toward a complex genetic architecture for lodging in wheat and ‘hidden heritability’ like the classic example of human height (Gudbjartsson et al., 2008).

### Genome-Wide Predictions and Cross-Validations

To address the postulate of hidden heritability due to a highly polygenic genetic architecture of numerous small-effect QTL, we generated genome-wide predictions for digital and visual measures of lodging. Three different genomic prediction models (BayesCπ, RKHS, and RR-BLUP) were compared to account for complex genetic architecture of lodging in wheat. As all three models yielded comparable results (Supplementary Table S3), only RR-BLUP based predictions are discussed. To assess the proportion of genetic variance captured using the whole-genome models, we calculated *k*-fold cross validation prediction accuracy within each year. Cross-validations within the environment were able to explain up to 28% of the heritable genetic effects (i.e., squared predictive ability) (Table 3). Finding that the whole genome models accounted for more than one-fourth of the heritable genetic effects for lodging, we further validated this observation by comparing the phenotypic and genetic correlation to the prediction accuracy for lodging measured in a second environment location of Faisalabad, Pakistan (FAS) (Supplementary Table S4). In majority of the cases the genomic prediction accuracies were equal or higher than the phenotypic correlation. In 2017 the whole-genome prediction model had a predictive correlation of 0.45, accounting for 20% of the heritable genetic effects for DLmix. Commensurate with application of genomic prediction in a breeding program with confounding environmental effects, the prediction accuracies across environments were lower but still captured heritable variance with predictions in the range of 0.19–0.55 (Table 4). In contrast to the lack of power to find individual genetic associations, we were able to capture a substantial portion of the heritable genetic effects using whole-genome models that account for many small effect QTL and support the hypothesis of a polygenic genetic architecture for lodging in elite wheat germplasm (Kooke et al., 2016). Furthermore, we support the observation that much larger populations must be evaluated to uncover the genetic basis and identify causative variants for lodging.

**Table 3:** 11-fold cross-validation predictive ability (*r*_pv_), broad-sense heritability (*H*^2^), and prediction accuracy (*r*_pa_) of visual and digital lodging measures in years 2016 and 2017 at LDH.

	2016			2017		
	*r*_pv_	*H*^2^	*r*_pa_	*r*_pv_	*H*^2^	*r*_pa_
LOI	0.30	0.59	0.39	0.41	0.67	0.50
LOS	0.31	0.50	0.44	0.41	0.63	0.52
LI	0.32	0.55	0.43	0.40	0.63	0.50
DLmean	0.35	0.56	0.47	0.40	0.71	0.47
DLmix	0.31	0.52	0.43	0.42	0.63	0.53

*r_pv_, correlation between genomic estimated breeding values and phenotypic values; r_pa_, predictive ability scaled to the squared root of heritability. LOI, lodging incidence; LOS, lodging severity; LI, lodging index; DLmean, digital lodging mean; DLmix, digital lodging mixture.*

**Table 4:** Prediction accuracies of lodging measures generated from different training-testing combinations on untested genotypes at LDH and FAS locations, e.g., 16LDH-17FAS is 16LDH training set predicting 17FAS.

	16LDH-17LDH	17LDH-16LDH	16LDH-17FAS	17LDH-16FAS	Average
LOI	0.37	0.45	0.28	0.27	0.34
LOS	0.42	0.54	0.32	0.23	0.38
LI	0.39	0.49	0.27	0.21	0.34
DLmean	0.37	0.51	0.30	0.20	0.35
DLmix	0.38	0.55	0.29	0.19	0.35

*LOI, lodging incidence; LOS, lodging severity; LI, lodging index; DLmean, digital lodging mean; DLmix, digital lodging mixture.*

### Relationship of Lodging to Phenology and Agronomic Traits

Finally, to investigate the relationship of different measures of lodging with plant developmental and agronomic traits, we calculated pairwise correlations of lodging with different traits within each environment. Consistent with previous reports in wheat (Keller et al., 1999; Berry and Berry, 2015), we found that taller plants (*r* = 0.12, *P* < 0.01) with early heading (*r* = -0.15, *P* < 0.001) tend to have more lodging while thousand grain weight was negatively associated with lodging (Supplementary Figure S4). A positive relationship of lodging and plant biomass traits such as ground coverage, plant stand, and spike length also highlights the vulnerability of high yielding, high biomass cultivars to the crop lodging. Inconsistent trends, however, in the relationship between grain yield and lodging were observed with a negative correlation in 2016 and a positive correlation in 2017. This suggests that on occasion higher grain weight on the maturing spike can weigh the plants down and increase lodging, while in other conditions the lodging can occur at a stage that will prevent completion of grain filling leading to yield loss. Under preferred mechanical harvesting operations in farmers’ fields, either of these situations will lead to economic loss of harvestable yield and decreased quality. These trends suggest the need for developing cultivars with better stem strength characteristics to mitigate lodging associated losses.

### Implementation of the Proposed Methodology in Field Experiments

The present study implemented an efficient and scalable approach to measure complex phenological trait of lodging in the field experiments. Data collection component of this phenotyping approach includes UAS setup (i.e., mission generation and upload, calibration info collection) and aerial image acquisition. The flight time depends on the field scale, UAS flying elevation, moving velocity, and the overlapping rate between the successive aerial images. Data processing component includes DEM generation by photogrammetry and plot-level data extraction, and the processing time depend on the data volume and the computer hardware settings. For the implementation standpoint, this work can be replicated with an initial investment of USD 12000, which will cover the cost of sensor hardware (USD 2000), UAS platform (USD 5000), a high-precision GNSS (USD 2000), and the computer software and hardware (USD 3000). A practical implementation of our lodging assessment approach in the field would require a careful monitoring of weather and crop growth conditions. Furthermore, as the operational costs and scale of breeding programs grow in future, the cost-effective and high-throughput tools that can provide multiple layers of data at a fraction of cost would be highly desired. Therefore, a full benefit of our proposed methodology can be realized by integrating it with the routine application of UAS-based trait measurements in research programs. As such, this lodging assessment approach can provide an additional data layer on top of the routine phenotypic measurements (e.g., spectral, morphological, physiological) without incurring any extra cost and time effort to the researchers.

## Conclusion

Unmanned aerial systems-enabled phenotyping allowed us to quantify lodging on 2,640 wheat plots. Using validated digital lodging measurements along with association and genomic prediction analyses, we provide evidence in support of a polygenic genetic architecture of lodging in wheat. Our findings have diverse applications in plant breeding and genetics. First, our highly reproducible UAS based digital lodging methods can be easily scaled and also applied to different crops to rapidly quantify plant height, lodging, and should be extensible to traits like growth rate on large populations. Second, for a complex and quantitatively controlled trait like lodging, whole-genome predictions can account for heritable variation not captured by regular GWAS. Undoubtedly, accurate phenotypic assessment is a critical prerequisite for breeding for lodging resilience, and as shown here, UAS-enabled large-scale quantitative assessment of lodging can be a powerful approach to identify genetic variants for lodging. This comprehensive evaluation of lodging assessment methods lays the foundation for improving our understanding of functional underpinnings of lodging in wheat and other crops. Overall this highlights the future of modern breeding where, in conjunction with powerful genomics and informatics tools, UAS-enabled phenotyping can accelerate the genetic gains in plant breeding to meet the global demand for food, fiber, and fuel.

## Author Contributions

JP and DS conceived and designed the study and wrote the manuscript. DS collected and analyzed UAS and ground-truth data in India. DS and XW performed image analysis. LG contributed alien-fragment data. UK supervised field experiments and collected data in India. MN and MI supervised field experiments and collected ground-truth data in Pakistan. RS provided experimental lines. JP directed the overall project. All authors edited and reviewed the manuscript.

## Conflict of Interest Statement

The authors declare that the research was conducted in the absence of any commercial or financial relationships that could be construed as a potential conflict of interest.

## Footnotes

1 http://ardupilot.org/planner/

2 https://croptsrv-cdc.it.csiro.au/shiny/users/zhe00a/missionplanner/

3 https://github.com/micasense/imageprocessing

4 https://doi.org/10.6084/m9.figshare.6151127
