# Supplementary material for: High-Throughput Phenotyping Enabled Genetic Dissection of Crop Lodging in Wheat
Source: Front Plant Sci. 2019 Apr 3;10:394. doi: 10.3389/fpls.2019.00394 (PMC6459080; doi:10.3389/fpls.2019.00394)
Supplement: Supplementary file 1 [file Data_Sheet_1.PDF]

Supporting Information

Article title: High-throughput phenotyping enabled genetic dissection of crop lodging in wheat

Authors: Daljit Singh, Xu Wang, Uttam Kumar, Liangliang Gao, Muhammad Noor, Muhammad Imtiaz, Ravi P. Singh, Jesse Poland

The following supporting information is available for this article:

Figure S1. Manhattan plot of genome-wide associations in 2016.

Figure S2. Manhattan plot of genome-wide associations in 2017.

Figure S3. Quantile-Quantile (QQ) plots of the combined genome-wide association analysis.

Figure S4. Pairwise relationship of lodging and agronomic traits in (A) year 2016, (B) year 2017. Diagonal panels show trait distributions; upper triangle is the Pearson's correlation coefficient with significance levels as superscript ($^{*}P < 0.05$; $^{**}P < 0.01$; $^{***}P < 0.001$); lower triangle is the scatter plot.

Table S1. Ontological description of phenotypic traits assessed during the study.

Table S2. Genetic correlations of lodging measures in years 2016 and 2017.

Table S3. Comparison of predictive abilities of three genomic prediction models.

Table S4. Predictive ability (r_{pv}), phenotypic correlation (r_{ph}) and genetic correlation (r_g) of lodging between LDH and FAS in years 2016 and 2017.

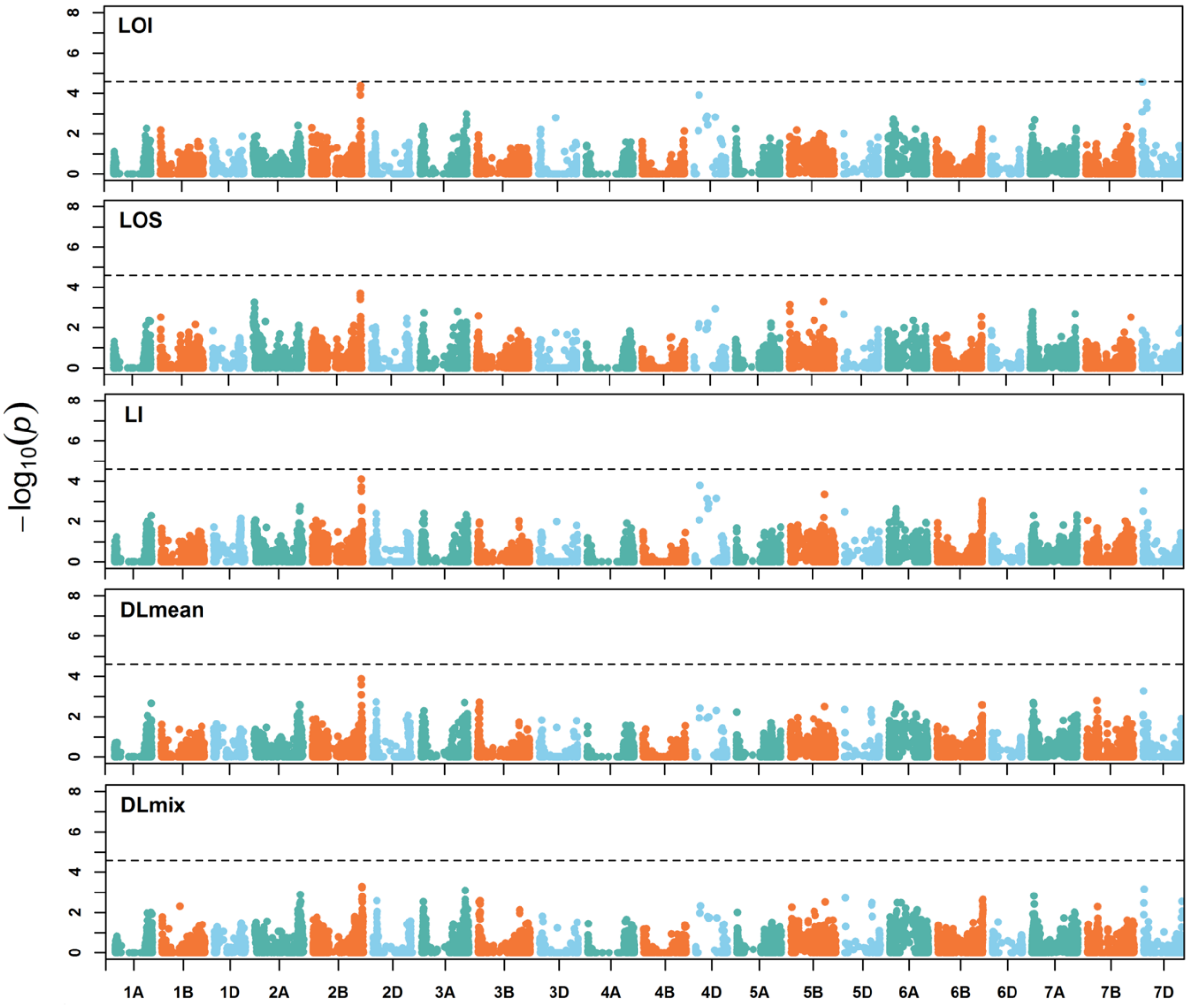

Figure S1. Manhattan plot of genome-wide associations. Manhattan plots of visual and digital lodging scores in season 2016. The dashed lines on y-axis correspond to the genome-wide false discovery rate (FDR) threshold.

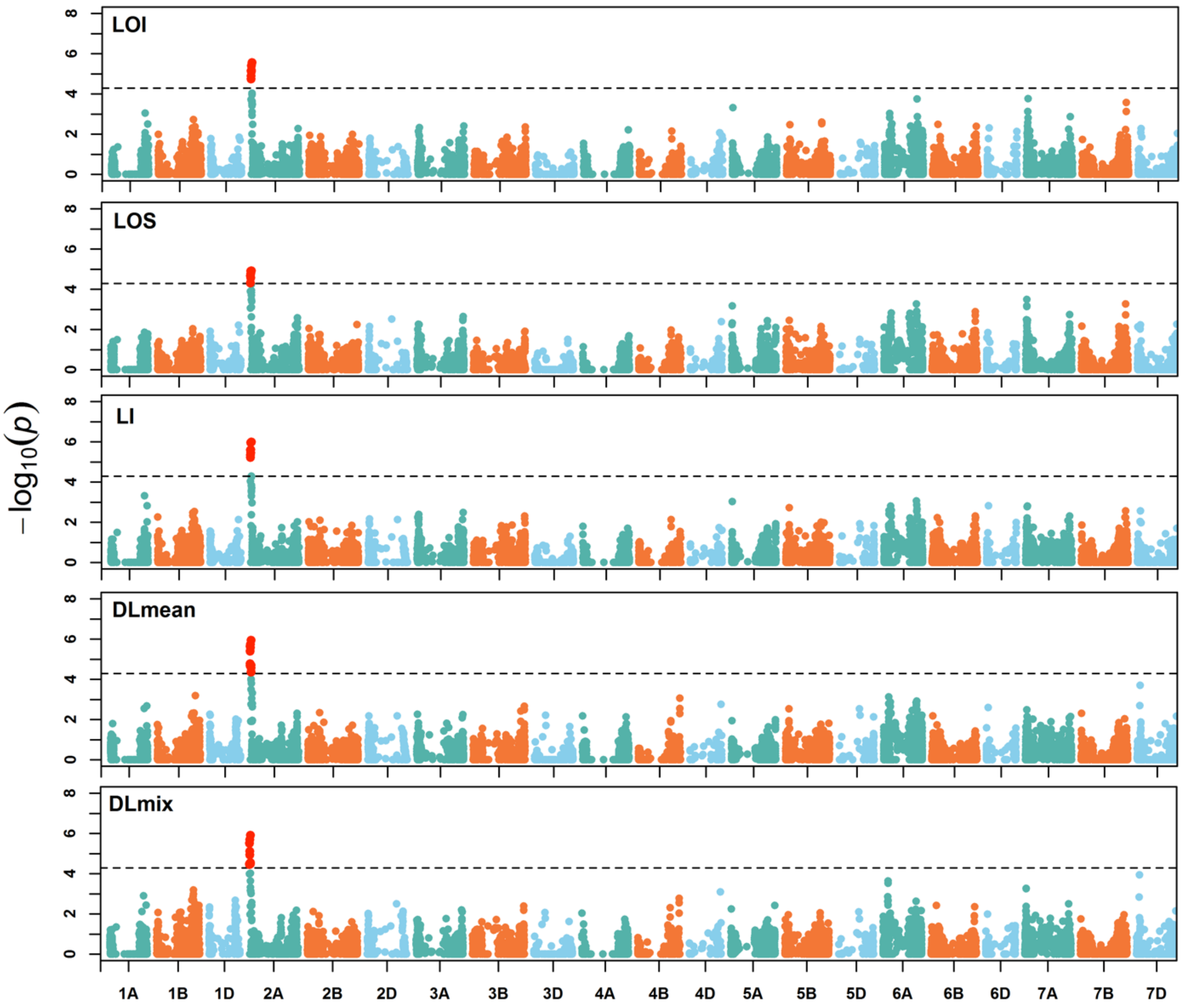

Figure S2. Manhattan plot of genome-wide associations. Manhattan plots of visual and digital lodging scores in season 2017. The dashed lines on y-axis correspond to the genome-wide false discovery rate (FDR) threshold.

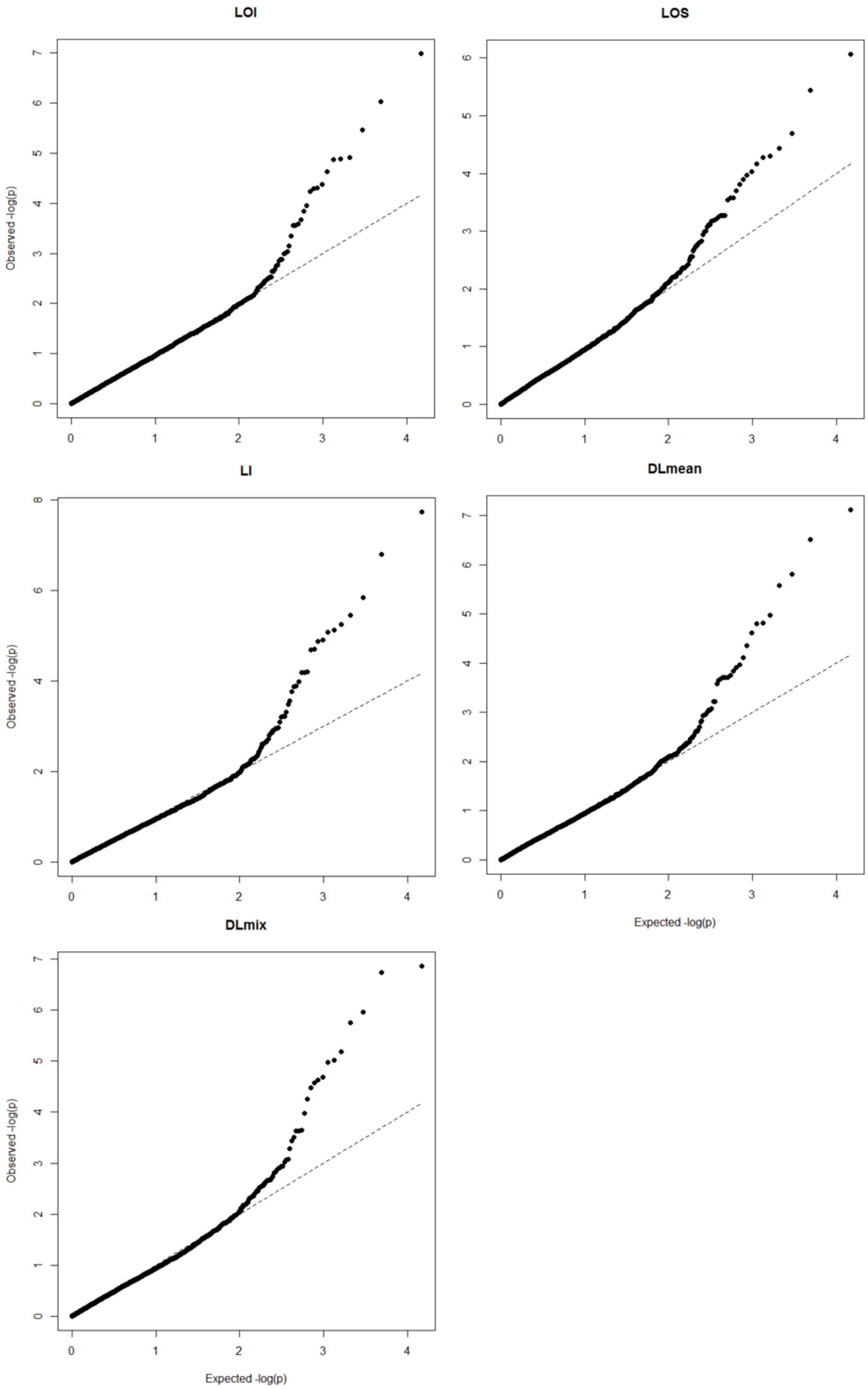

Figure S3. Quantile-Quantile (QQ) plots of the genome-wide associations. The QQ plots of SNP markers of the measured lodging traits.

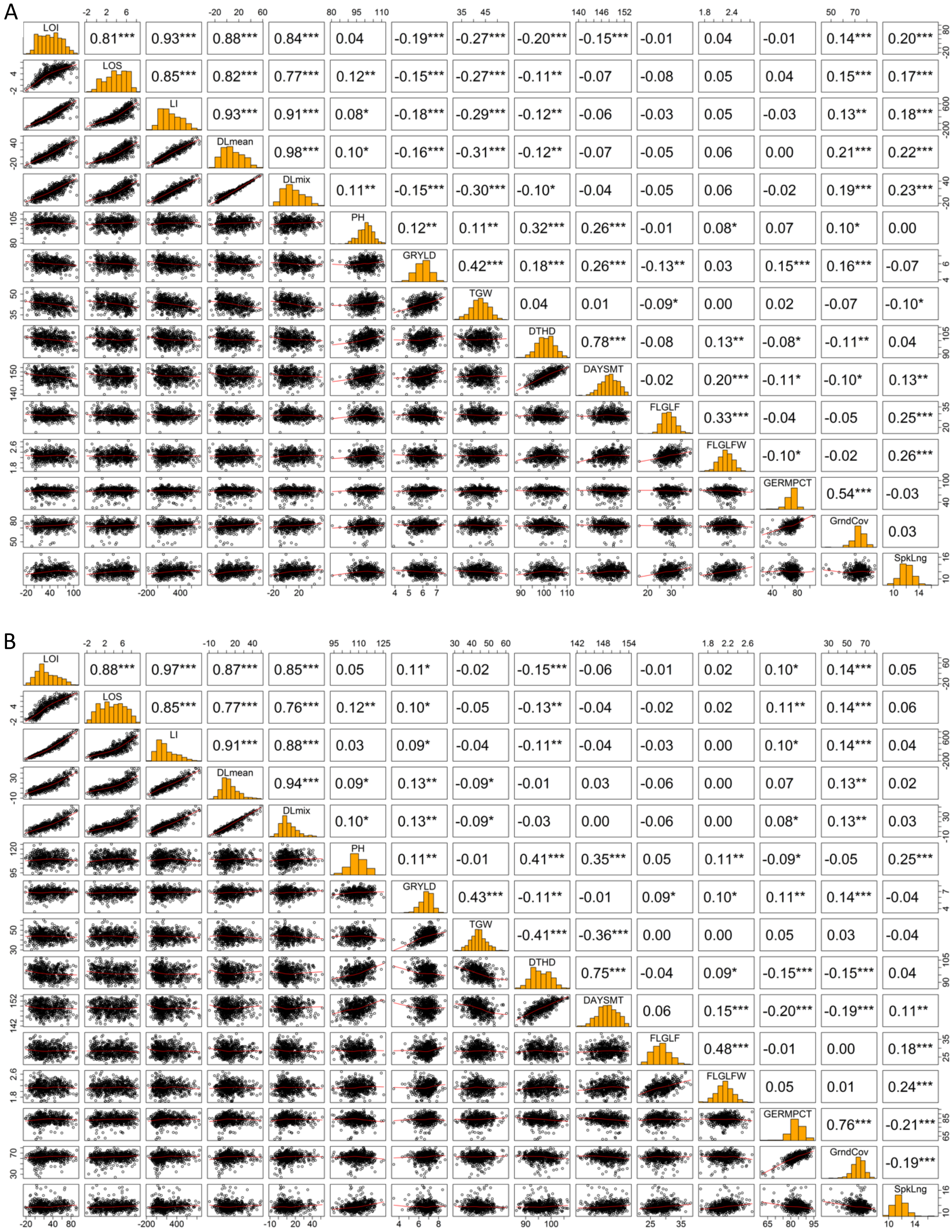
A
LOI 0.81*** 0.93*** 0.88*** 0.84*** 0.04 -0.19*** -0.27*** -0.20*** -0.15*** -0.01 0.04 -0.01 0.14*** 0.20***
LOS 0.85*** 0.82*** 0.77*** 0.12** -0.15*** -0.27*** -0.11** -0.07 -0.08 0.05 0.04 0.15*** 0.17***
LI 0.93*** 0.91*** 0.08* -0.18*** -0.29*** -0.12** -0.06 -0.03 0.05 -0.03 0.13** 0.18***
DLmean 0.98*** 0.10* -0.16*** -0.31*** -0.12** -0.07 -0.05 0.06 0.00 0.21*** 0.22***
DLmix 0.11** -0.15*** -0.30*** -0.10* -0.04 -0.05 0.06 -0.02 0.19*** 0.23***
PH 0.12** 0.11** 0.32*** 0.26*** -0.01 0.08* 0.07 0.10* 0.00
GRYLD 0.42*** 0.18*** 0.26*** -0.13** 0.03 0.15*** 0.16*** -0.07
TGW 0.04 0.01 -0.09* 0.00 0.02 -0.07 -0.10*
DTHD 0.78*** -0.08 0.13** -0.08* -0.11** 0.04
DAYSMT -0.02 0.20*** -0.11* -0.10* 0.13**
FLGLF 0.33*** -0.04 -0.05 0.25***
FLGLFW -0.10* -0.02 0.26***
GERMPCT 0.54*** -0.03
GrndCov 0.03
SpkLng
B
LOI 0.88*** 0.97*** 0.87*** 0.85*** 0.05 0.11* -0.02 -0.15*** -0.06 -0.01 0.02 0.10* 0.14*** 0.05
LOS 0.85*** 0.77*** 0.76*** 0.12** 0.10* -0.05 -0.13** -0.04 -0.02 0.02 0.11** 0.14*** 0.06
LI 0.91*** 0.88*** 0.03 0.09* -0.04 -0.11** -0.04 -0.03 0.00 0.10* 0.14*** 0.04
DLmean 0.94*** 0.09* 0.13** -0.09* -0.01 0.03 -0.06 0.00 0.07 0.13** 0.02
DLmix 0.10* 0.13** -0.09* -0.03 0.00 -0.06 0.00 0.08* 0.13** 0.03
PH 0.11** -0.01 0.41*** 0.35*** 0.05 0.11** -0.09* -0.05 0.25***
GRYLD 0.43*** -0.11** -0.01 0.09* 0.10* 0.11** 0.14*** -0.04
TGW -0.41*** -0.36*** 0.00 0.00 0.05 0.03 -0.04
DTHD 0.75*** -0.04 0.09* -0.15*** -0.15*** 0.04
DAYSMT 0.06 0.15*** -0.20*** -0.19*** 0.11**
FLGLF 0.48*** -0.01 0.00 0.18***
FLGLFW 0.05 0.01 0.24***
GERMPCT 0.76*** -0.21***
GrndCov -0.19***
SpkLng

Figure S4. Pairwise relationship of lodging and agronomic traits in (A) year 2016, (B) year 2017. Diagonal panels show trait distributions; upper triangle is the Pearson's correlation coefficient with significance levels as superscript ($^{*}P < 0.05$; $^{**}P < 0.01$; $^{***}P < 0.001$); lower triangle is the scatter plot.

Table S1. Description of phenotypic traits assessed during field experiments

Trait	Abbreviation	Units	Ontology	Description
Ground Cover	GrndCov	%	CO_321:0000014	Crop ground cover, or the percentage of soil surface covered by plant foliage
Grain Yield	GRYLD	t ha^{-1}	CO_321:0000013	Amount (weight) of grains that was harvested
Plant Height	PH	cm	CO_321:0000020	Height of plant from ground to top of spike, excluding awns
Thousand Grain Weight	TGW	g	CO_321:0000025	Grain weight expressed as weight of a thousand grains
Heading Date	DTHD	-	CO_321:0000007	Heading time extends from the time of emergence of the tip of the spike from the flag leaf sheath to when the spike has completely emerged but has not yet started to flower
Days to Maturity	DAYSMT	-	CO_321:0000022	Maturity time starts at hard dough stage (ds87) often called physiological maturity

Flag Leaf Length	FLGLF	cm	CO_321:0001524	Length of flag leaf lamina
Flag Leaf Width	FLGLFW	cm	CO_321:0001525	Width of flag leaf lamina
Germination Percentage	GERMPCT	%	CO_321:0000011	Proportion of seed that germinate under proper condition
Spike Length	SpkLng	cm	CO_321:0000056	Length of spike.
Lodging Incidence	LOI	%	CO_321:0000167	Indicates incidence of lodged plants
Lodging Severity	LOS	-	-	Severity of the lodged plants on 0 to 10 scale

Table S2. Genetic correlations of lodging measures in years 2016 and 2017. Lower triangle (2016); upper triangle (2017). LOI, lodging incidence; LOS, lodging severity; LI, lodging index (LOI × LOS); DLmean, digital lodging mean; DLmix, digital lodging mixture.

Phenotype	LOI	LOS	LI	DLmean	DLmix
LOI	-	0.98	0.99	0.95	0.96
LOS	0.93	-	0.96	0.93	0.93
LI	0.93	0.99	-	0.96	0.97
DLmean	0.94	0.96	0.96	-	1.00
DLmix	0.93	0.96	0.97	1.00	-

Table S3. Comparison of predictive abilities of three genomic prediction models (RR: RRBLUP; BCπ: Bayesian Cπ; RKHS: Reproducing Kernel Hilbert Space) of visual and digital lodging measures. Tr: Training set; Pr: Prediction set.

	Tr16-Pr17			*Tr17-Pr16*		
	RR	*BCπ*	*RKHS*	*RR*	*BCπ*	*RKHS*
LOI	0.30	0.30	0.30	0.32	0.32	0.33
LOS	0.35	0.35	0.35	0.38	0.37	0.38
LI	0.32	0.32	0.32	0.35	0.35	0.35
DLmean	0.32	0.31	0.32	0.37	0.37	0.37
DLmix	0.32	0.31	0.32	0.39	0.39	0.39

Table S4. Predictive ability (r_{pv}), phenotypic correlation (r_{ph}) and genetic correlation (r_g) of lodging between LDH and FAS in years 2016 and 2017.

	2016			*2017*		
	r_{pv}	r_{ph}	r_g	r_{pv}	r_{ph}	r_g
LOI	0.43	0.39	0.65	0.45	0.46	0.75
LOS	0.32	0.29	0.45	0.46	0.44	0.88
LI	0.36	0.33	0.54	0.44	0.44	0.77
DLmean	0.33	0.32	0.54	0.41	0.42	0.66
DLmix	0.30	0.29	0.51	0.40	0.41	0.67

r_{pv}: correlation between genomic estimated breeding values and phenotypic values across environments

r_{ph}: correlation of phenotypic values at two environments

r_g: the marker-based genetic correlation between two environments
